# Lactate Suppresses Retroviral Transduction in Cervical Epithelial Cells through DNA-PKcs Modulation

**DOI:** 10.3390/ijms222413194

**Published:** 2021-12-07

**Authors:** Waldemar Wagner, Katarzyna Sobierajska, Katarzyna Dominika Kania, Edyta Paradowska, Wojciech Michał Ciszewski

**Affiliations:** 1 Laboratory of Cellular Immunology, Institute of Medical Biology PAS, 106 Lodowa Street, 93-232 Lodz, Poland; 2 Department of Molecular Cell Mechanisms, Medical University of Lodz, Mazowiecka 6/8 Street, 92-215 Lodz, Poland; katarzyna.sobierajska@umed.lodz.pl (K.S.); wojciech.ciszewski@umed.lodz.pl (W.M.C.); 3 Laboratory of Virology, Institute of Medical Biology PAS, 106 Lodowa Street, 93-232 Lodz, Poland; kkania@cbm.pan.pl (K.D.K.); eparadow@cbm.pan.pl (E.P.)

**Keywords:** lactate, DNA repair, NU7441, DNA-PKcs, lentivirus, HCA1, HDAC, MCT, BAY-8002, cervical cancer

## Abstract

Recently, we have shown the molecular basis for lactate sensing by cervical epithelial cells resulting in enhanced DNA repair processes through DNA-PKcs regulation. Interestingly, DNA-PKcs is indispensable for proper retroviral DNA integration in the cell host genome. According to recent findings, the mucosal epithelium can be efficiently transduced by retroviruses and play a pivotal role in regulating viral release by cervical epithelial cells. This study examined the effects of lactate on lentiviral transduction in cervical cancer cells (HeLa, CaSki, and C33A) and model glioma cell lines (DNA-PKcs proficient and deficient). Our study showed that L- and D-lactate enhanced DNA-PKcs presence in nuclear compartments by between 38 and 63%, which corresponded with decreased lentiviral transduction rates by between 15 and 36%. Changes in DNA-PKcs expression or its inhibition with NU7441 also greatly affected lentiviral transduction efficacy. The stimulation of cells with either HCA1 agonist 3,5-DHBA or HDAC inhibitor sodium butyrate mimicked, in part, the effects of L-lactate. The inhibition of lactate flux by BAY-8002 enhanced DNA-PKcs nuclear localization which translated into diminished lentiviral transduction efficacy. Our study suggests that L- and D-lactate present in the uterine cervix may play a role in the mitigation of viral integration in cervical epithelium and, thus, restrict the viral oncogenic and/or cytopathic potential.

## 1. Introduction

Vaginal and ectocervical microbiota of the female genital tract (FGT) protects against pathogen colonization through the competition for adherence, the production of antimicrobial substances, and the secretion of high amounts of lactate. Vaginal secretions may contain from 10–50 mM lactate; approximately 55% of vaginally secreted lactate is the D isoform [1]. Recently, we have reported the lactate-driven mechanisms by which lactate under physiological conditions modulates the activity of DNA repair machinery of the cervical epithelial cells. These pathways include the stimulation of surface-specific lactate hydroxycarboxylic acid receptor 1 (HCA1/GPR81) and the decrease of nuclear chromatin compactness by the inhibition of HDAC (histone deacetylase) [2,3]. HCA1 belongs to the G-protein-coupled receptor family, and when coupled to a Gi-protein, initiates two downstream signaling cascades (cAMP and Erk1/2 pathways) upon activation by its natural ligands: L-lactate, D-lactate, or dihydroxybenzoic acid (DHBA) [2,4]. So far, surface HCA1 has been implicated in the regulation of cancer growth, metastasis, neuronal activity, lipids metabolism, energy regulation, immune tolerance, wound healing, and more [5,6,7]. Inside cells, lactate exhibits weak inhibitory activity against histone deacetylases (HDAC) [2,8] resulting in histones hyperacetylation and a more relaxed, transcription, and repair-proficient chromatin state that orchestrates the activity of repair and signaling proteins. Cellular lactate influx/efflux is dependent on the activity of monocarboxylate transporters (MCTs 1–4), directed by substrate and proton concentration gradients [9].

Our former study revealed that both isomers, L- and D-lactate, enhanced DNA repair mechanisms in cervical cancer cells via a lactate-elicited increase in activity of the DNA-PK catalytic subunit (DNA-PKcs) and/or its enhanced nuclear localization [2,10]. DNA-PKcs is an abundant protein directly mediating the ligation of the broken ends of the DNA double-strand breaks, following the recognition and binding of the broken DNA ends by the Ku70/Ku80 protein heterodimer. Thus, DNA-PKcs is the essential component of the nonhomologous end-joining pathway (NHEJ) involved in cellular DNA damage response [11]. It is widely accepted that DNA-PKcs is a pleiotropic enzyme that possesses a variety of functionalities outside of NHEJ. Due to the ability of DNA-PKcs to sense foreign or damaged self-DNA, the NHEJ pathway components have extensive interactions with numerous different viruses, including dsDNA viruses: ADV, HPV, HSV-1, EBV, and ssRNA-RT viruses: HIV and HTLV [12]. The role of DNA-PKcs in virus integration was originally suggested by Daniel et al. [13]. This theory was initially questioned [14], and now, the accumulating evidence indicates the essential role of DNA-PKcs and its kinase activity in the post-integrational DNA repair of lentiviral infections (such as HIV-1) [15,16]. Apart from its kinase activity, DNA-PKcs could also interact with cyclin T1, cyclin-dependent kinase 9, and Tat (HIV-1 transactivator of transcription), acting as a scaffold protein leading to the inhibition of HIV-1 transcription [17]. 

The mucosal epithelia of the FGT are the first contact site between viruses and the human body during infection [18]. The cervical epithelium barrier is frequently compromised by sexually transmitted infections and has been suggested to be the site of high susceptibility and initial oncogenic (HPV) and cytopathic (HIV-1) viral infections during sexual transmission [19]. On the other hand, it has been shown that the mucosal epithelium, in addition to serving as a natural specific barrier against pathogens, may also be actively involved in the transmission of HIV-1 infection. Cervical epithelial cells are widely believed to support internalizing, transcytosing, and transinfecting CD4-positive cells with HIV-1 [20,21]. The majority of reports indicate that this process takes place without infection of the epithelial cells, the recent reports emphasize that these cells are also susceptible to productive infection with HIV-1 [22,23,24]. The most current reports indicated that bronchial, renal, and gastric epithelial cells could also produce infectious retroviruses as a result of viral transcription and integration [25,26,27,28]. Nevertheless, the consensus among scientists has not been reached yet about HIV transmission in other cells than immune cells and such interpretations remain ambiguous. 

The aim of the present study was to assess the role of lactate in the modulation of DNA-PKcs, which play a crucial role in cellular DNA repair as well as an indispensable role in retroviral DNA integration. Since cervical epithelial cells are prone to the modulatory action of lactobacilli-derived lactate we decided to evaluate the transduction efficacy of engineered retroviral vectorsin relation to the cellular localization of DNA-PKcs in human cervical epithelial cancer cells exposed to the physiological concentration of lactate. The present study reports a novel biological activity of lactate, specifically in the modulation of transduction efficacy of lentivirus in cervical cancer cells. Our study indicates that lactate present in the uterine cervix may play a role in the mitigation of viral integration in the cervical epithelium and, thus, restrict the viral oncogenic and/or cytopathic potential.

## 2. Results

### 2.1. L- and D-Lactate Stimulate DNA-PKcs Nuclear Translocation in Cancer Cervical Epithelial Cells

**Figure 1:**
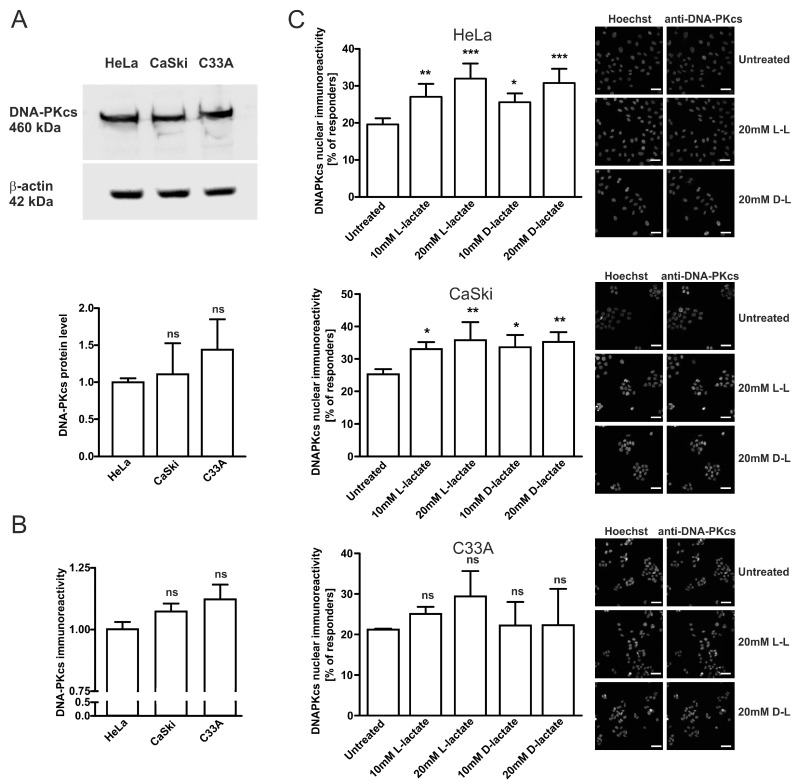
Lactate enhances nuclear localization of DNA-PKcs in cervical cancer cell lines. (**A**) The DNA-PKcs protein level in cell extracts was quantified using densitometry and presented as relative means ± SD of DNA-PKcs/β-actin ratio normalized to HeLa cells (n = 4). (**B**) Immunocytochemical staining of DNA-PKcs, results are presented as relative means ± SD normalized to HeLa cells (n = 4), calculated from four independent experiments. (**C**) Cervical cancer cells were treated with increasing concentrations of L-lactate (L-L) and D-lactate (D-L) for 24 h before measuring cellular DNA-PKcs immunoreactivity. The graphs display the percentage of high DNA-PKcs immunoreactive cells (responders) of HeLa, CaSki, and C33A cells whose fluorescence overcame the threshold of mean fluorescence set on untreated corresponding cells. Data are presented as mean ± SD. Statistical significance was evaluated using one-way ANOVA followed by Tukey’s test. * *p* < 0.05, ** *p* < 0.01 and *** *p* < 0.001 indicate significant; not significant, ns. The scale bar is 50 µm.

Previously, we have shown that lactate enhances DNA-PKcs activation in neocarzinostatin-treated HeLa cells [2]. Interestingly, the lactate-driven enhancement of DNA-PKcs activation was also accompanied by higher DNA-PKcs nuclear immunoreactivity, indicating the increased localization of the protein in the nucleus [10]. This study examined the DNA-PKcs level in cervical cancer cells: HeLa, CaSki, and C33A. The lowest protein level was attributed to the HeLa cells, while CaSki and C33A exhibited a 1.11- and 1.44-fold higher DNA-PKcs level than HeLa, respectively (Figure 1A). Although the modulations of the DNA-PKcs level were not statistically significant, its level in CaSki and C33A cell lines showed an upward trend. A similar pattern was observed when DNA-PKcs nuclear translocation using the immunocytochemistry technique was examined (Figure 1B). Then, we evaluated the effects of L- and D-lactate on DNA-PKcs nuclear translocation. After 24 h incubation of HeLa cells with 10 mM or 20 mM of L-lactate, we observed 1.38- and 1.63-fold more cells exhibiting high DNA-PKcs immunoreactivity (responders), respectively, compared to untreated HeLa cells. After D-lactate stimulation, the percentage of responders was slightly lower and constituted for a 1.3- and 1.57-fold increase in cells responding to 10 mM and 20 mM treatments, respectively. A similar pattern of response after lactate treatment was observed for CaSki cells. Additionally, C33A cells showed increased nuclear DNA-PKcs immunoreactivity only after L-lactate stimulation. However, changes were not statistically significant (1.18- and 1.38-fold more responding cells for 10 mM and 20 mM L-lactate treatments, respectively). Incubation with D-lactate did not affect DNA-PKcs nuclear localization in C33A cells. 

### 2.2. L- and D-Lactate Suppress Lentiviral Transduction in Cancer Cervical Epithelial Cells

**Figure 2:**
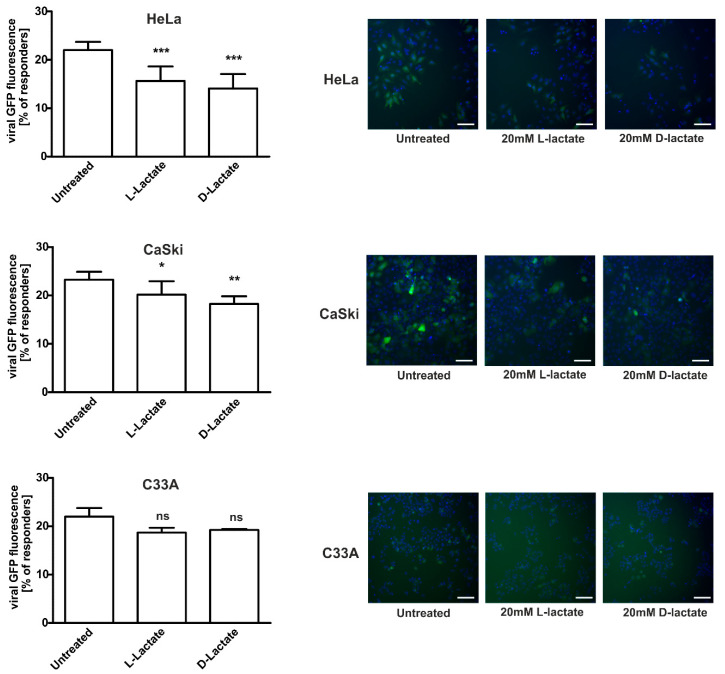
L- and D-lactate treatment affect the transduction efficacy of lentivirus. Cervical cancer cells were incubated in the presence or absence of 20 mM L- or D-lactate for 24 h, followed by infection with copGFP Control Lentiviral Particles at MOI = 5 for 24 h before changing the medium. After 48 h, the infected cells were stained with Hoechst 33342 and immediately analyzed using an ArrayScan VTI HCS Reader. The graphs show the percentage of high copGFP expressing HeLa, CaSki, and C33A cells (responders) whose fluorescence overcame the threshold of mean fluorescence set on untreated cells. Data are presented as mean ± SD (n = 3). Images showed representative microscopic areas for the particular treatment from the same experiment. * *p* < 0.05, ** *p* < 0.01 and *** *p* < 0.001; not significant, ns. The scale bar is 100 µm.

Recently, it has been proposed that DNA-PK is essential for HIV-1 infectivity and post-integrational DNA gap repair [13]. Since lactate triggers DNA-PKc nuclear retention, we investigated whether lactate would affect the efficacy of lentiviral transduction in cervical cancer cells. Following 24 h-treatment with 20 mM of either lactate isomer, we infected epithelial cells with lentiviral particles containing a copGFP coding construct for expression in mammalian cells. Effectively transduced cells, responders, were characterized by intense GFP fluorescence. After 48 h of incubation, we observed statistically significant reduced lentivirus transduction efficacy in HeLa cells treated with both lactate isomers (Figure 2). L-lactate and D-lactate treatments caused a 0.71- and 0.64-fold reduction of lentivirus transduction within HeLa cells, respectively. Incubating CaSki cells with L-lactate and D-lactate lactate exerted similarly but slightly lower effects as observed for HeLa, being a 0.87- and 0.78-fold reduction of lentivirus transduction, respectively. (Figure 2). The inhibitory effect of lactate on lentivirus transduction efficacy in C33A cells was the lowest among the tested cell lines (Figure 2). We observed the non-statistically significant downward trend. Both L- and D-lactate treatments reduced lentivirus transduction 0.85-fold, but the observed effect was not statistically significant. 

### 2.3. DNA-PKcs-Proficient Glioma Cells Are Prone to Lactate-Driven Modulation of DNA-PKcs and Lentivirus Transduction Efficacy but Not DNA-PKcs-Deficient Counterpart

**Figure 3:**
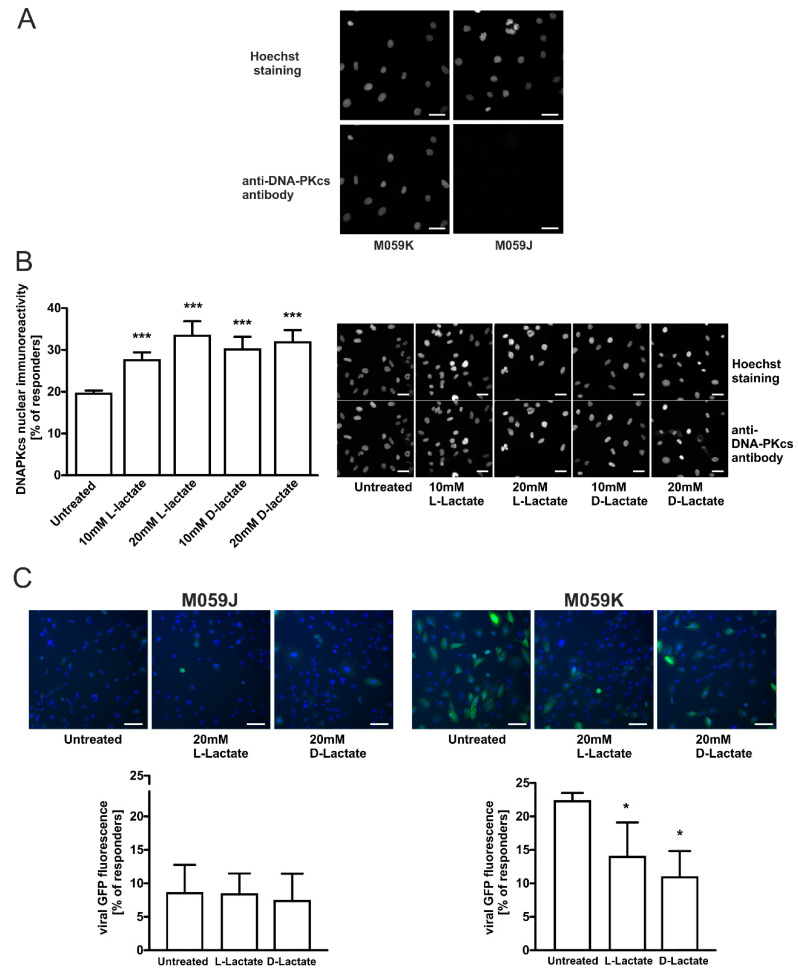
DNA-PKcs-proficient glioma M059K cells are prone to lactate-driven modulation of DNA-PKcs and lentivirus transduction efficacy but not the DNA-PKcs-deficient counterpart-M059J. (**A**) Immunocytochemical staining of DNA-PKcs in M059K and M059J cells, representative images are shown. (**B**) M059K cells were treated with increasing concentrations of L-lactate and D-lactate for 24 h before measuring cellular DNA-PKcs immunoreactivity. The graphs display the percentage of high DNA-PKcsimmunoreactive M059K cells (responders) whose fluorescence overcame the threshold of mean fluorescence set on untreated cells.Data are presented as mean ± SD (n = 3). Scale bar is 50 µm (**C**) M059K and M059J cells were incubated in the presence or absence of 20 mM L- or D-lactate for 24 h, followed by infection with copGFP Control Lentiviral Particles at MOI = 5 for 24 h before changing the medium. After 48 h, the infected cells were stained with Hoechst 33,342 and immediately analyzed using an ArrayScan VTI HCS Reader. The graphs show the percentage of high copGFP expressing M059K and M059J cells (responders) whose fluorescence overcame the threshold of mean fluorescence set on untreated M059K cells. Data are presented as mean ± SD (n = 3). Images showed representative microscopic areas for the particular treatment from the same experiment. * *p* < 0.05, and *** *p* < 0.001. The scale bar is 100 µm.

To confirm the indispensable role of DNA-PKcs in the transduction of lentiviral particles and the modulatory effects of lactate on transduction efficacy, we employed an established model of glioma cells exhibiting the proficient and deficient DNA-PKcs phenotype, M059K and M059J, respectively. Using the immunocytochemical method, we confirmed that M059J cells were negatively immunostained with anti-DNA-PKcs antibodies (Figure 3A). Contrarily, M059K glioma cells exhibited strong DNA-PKcs immunoreactivity and responsiveness to lactate treatment. Both lactate isomers statistically significantly enhanced the nuclear localization of DNA-PKcs. After 24 h incubation of M059K cells with 10 mM or 20 mM of L-lactate, we observed 1.41- and 1.71-fold more cells exhibiting high DNA-PKcs immunoreactivity, respectively (Figure 3B). D-lactate treatment at 20 mM concentration also evoked a 1.63-fold increase in the percentage of responders. Subsequently, we evaluated the functional relevance of DNA-PKcs to the lactate-mediated repression of lentivirus transduction. While DNA-PKcs-proficient glioma M059K cells were found to be prone to lentiviral transduction at a rate of 22% of positively transduced cells, its DNA-PKcs-deficient counterpart, M059J, was profoundly defective in lentivirus transduction, and only 8.5% of the cell population was infected (untreated M059K vs. untreated M059J, *p* < 0.0001; Figure 3C). Furthermore, M059K cells were susceptible to the inhibition of lentiviral transduction by L- and D-lactate. The highest dose of D-lactate resulted in a statistically significant reduction (0.58-fold) of lentivirus transduction within M059K cells.

### 2.4. Specific DNA-PKcs Inhibitor NU7441 Affects Lentivirus Transduction Efficacy

**Figure 4:**
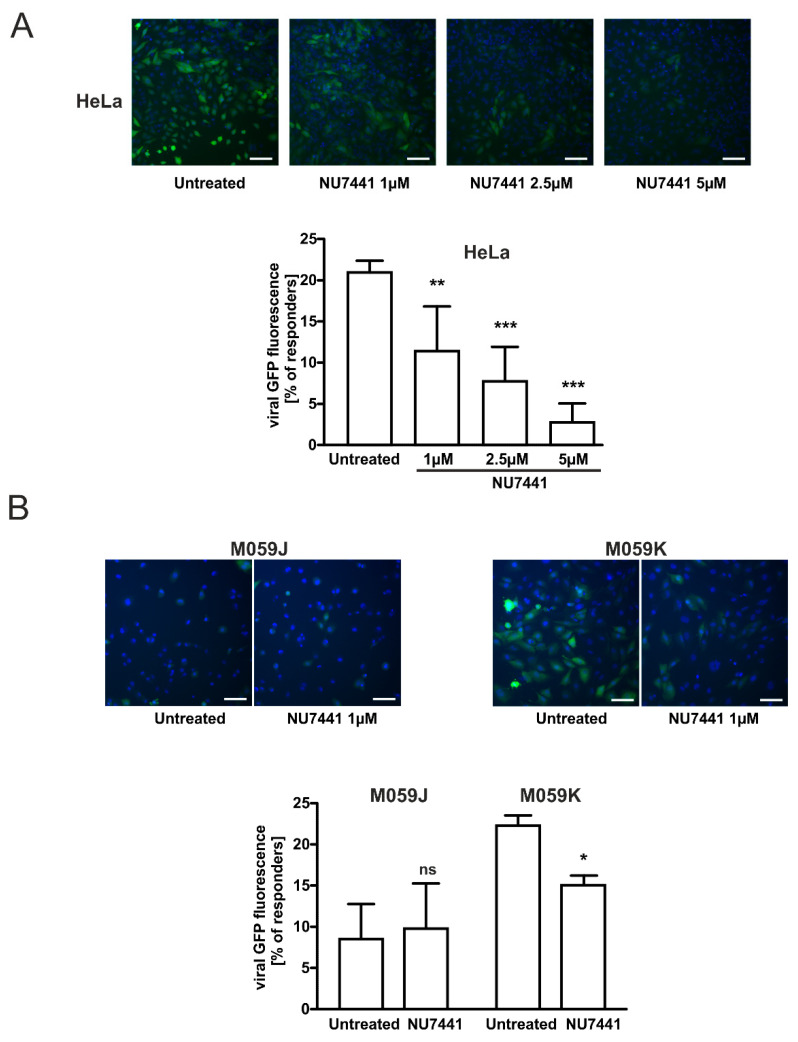
Specific DNA-PKcs inhibitor NU7441 diminishes lentivirus transduction efficacy. (**A**) HeLa cells were incubated in the presence or absence of 1, 2.5, and 5 µM NU7441 for 1 h followed by infection with copGFP Control Lentiviral Particles at MOI = 5 for 24 h before changing the medium. After 48 h, the infected cells were stained with Hoechst 33342 and immediately analyzed using an ArrayScan VTI HCS Reader. (**B**) M059K and M059J cells were incubated in the presence or absence of 1 µM NU7441 for 1 h followed by infection with copGFP Control Lentiviral Particles at MOI = 5 for 24 h before changing the medium. After 48 h, the infected cells were stained with Hoechst 33342 and immediately analyzed using an ArrayScan VTI HCS Reader. The graphs show the percentage of high copGFP expressing HeLa and M059K cells (responders) whose fluorescence overcame the threshold of mean fluorescence set on corresponding untreated cells. Data are presented as mean ± SD (n = 3). Images showed representative microscopic areas for the particular treatment from the same experiment. * *p*< 0.05, ** *p*< 0.01 and *** *p*< 0.001; not significant, ns. The scale bar is 50 µm.

It has been suggested that DNA-PKcs is involved in the post-integrational DNA repair step of the HIV-1 life cycle [15]. Moreover, Gottikh’s group recently showed that the inhibition of the phosphorylation activity of DNA-PKcs decreased the infectivity of HIV-1 in cells [15,29]. These results clearly show that the activity of DNA-PKcs is required for the efficient HIV-1 post-integrational gap repair and viral infectivity in different cell lines. Thus, to confirm the role of DNA-PKcs in the observed effects of lactate on lentivirus transduction efficacy, we performed experiments using a specific DNA-PKcs inhibitor NU7441. We detected a dose-dependent statistically significant inhibition of lentivirus transduction in HeLa cells treated with NU7441 (1–5 µM) for 1 h before cell infection (Figure 4A). NU7741 at the highest concentration used (5 µM) almost completely reduced the transduction efficacy rate in HeLa cells (by 0.13-fold). The efficacy of lentivirus transduction was also compromised in DNA-PKcs-proficient glioma M059K cells treated with 1 µM of NU7441. The transduction efficacy dropped significantly (0.72-fold) in pretreated inhibitor cells (Figure 4B). Thus, the combined results of our experiments indicate that any changes within the expression, activity, or compartmentalization of DNA-PKcs may affect the efficacy of viral cDNA integration as reported earlier [15].

### 2.5. HCA1 and HDAC Activities Are Required for Lactate-Mediated Enhancement of DNA-PKcs Nuclear Localization in HeLa Cells

To further explore how lactate mediates the enhancement of DNA-PKcs nuclear localization, we employed a specific HCA1 agonist, 3,5-dihydroxybenzoic acid (DHBA), and known HDAC inhibitor, sodium butyrate (NaB). Previously, we have shown that HCA1 stimulation or HCA1 restriction produced changes in the DNA-PKcs cellular compartmentalization in cervical cancer epithelial cells [10]. On the other hand, lactate was reported to exert its anti-HDAC activity by the induction of histones hyperacetylation and decrease of chromatin compactness at a comparable level as sodium butyrate [2]. Thus, in the next step, we evaluated the effects of DHBA (HCA1 agonist), sodium butyrate, and lactate to confirm whether the observed stimulatory effects of lactate on DNA-PKcs depend on extracellular lactate action (through HCA1 stimulation), intracellular lactate action (through HDAC inhibition), or both.

**Figure 5:**
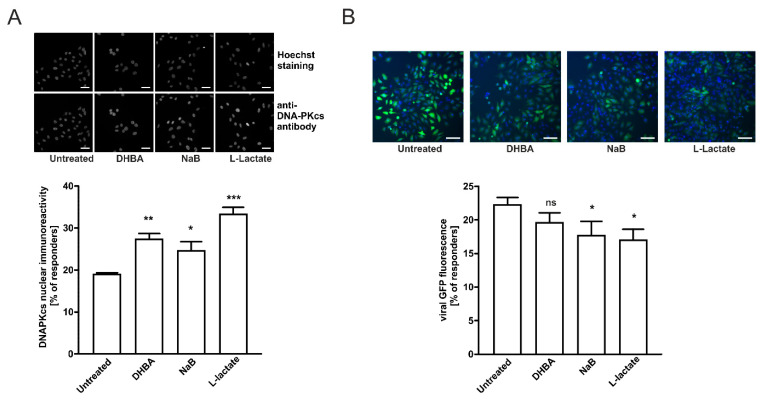
HCA1 and HDAC activity is required for lactate-mediated enhancement of DNA-PKcs nuclear localization and accounts for inhibition of lentiviral transduction in HeLa cells. (**A**) HeLa cells were treated with 250 µM DHBA, 0.5 mM sodium butyrate or 20 mM L-lactate for 24 h before measuring cellular DNA-PKcs immunoreactivity. The graphs display the percentage of high DNA-PKcs immunoreactive cells (responders) whose fluorescence overcame the threshold of mean fluorescence set on untreated cells. Data are presented as mean ± SD (n = 3) (**B**) HeLa were incubated in the presence or absence of 250 µM DHBA, 0.5 mM sodium butyrate, or 20 mM L-lactate, followed by infection with copGFP Control Lentiviral Particles at MOI = 5 for 24 h before changing the medium. After 48 h, infected cells were stained with Hoechst 33342 and immediately analyzed using an ArrayScan VTI HCS Reader. The graphs show the percentage of high copGFP expressing HeLa cells (responders) whose fluorescence overcame the threshold of mean fluorescence set on untreated cells. Data are presented as mean ± SD (n = 3). Images showed representative microscopic areas for the particular treatment from the same experiment. The scale bar is 50 µm. * *p* < 0.05, ** *p* < 0.01 and *** *p* < 0.001; not significant, ns.

To evaluate the possible role of HCA1 in L-lactate effects on nuclear DNA-PKcs, we stimulated HeLa cells with 250 µM DHBA or 0.5 mM NaB and compared them to cells stimulated with 20 mM L-lactate. Stimulating cells with 250 µM DHBA for 24 h resulted in enhanced DNA-PKcs nuclear localization observed in 27.3% of the cells compared to 18.95% of the untreated cells (Figure 5A). In both cases, the observed changes were statistically significant. Treatment with 0.5 mM sodium butyrate also increased the percentage of highly DNA-PKcs immunoreactive cells up to 24.6%. Interestingly, the net sum of particular treatment effects of DHBA (8.4%) and sodium butyrate (5.6%) constituted the total net effect observed for 20 mM L-lactate (14.2%). In the next step, we performed complementary experiments to assess the functional relevance of HCA1 and HDACs to the lactate-mediated repression of lentivirus transduction. Stimulating cells with 250 µM DHBA or 0.5 mM of sodium butyrate for 24 h resulted in the moderate reduction of lentivirus transduction efficacy in both DHBA- and sodium butyrate-treated HeLa cells (Figure 5B). The percentage of responders for DHBA was not statistically significant (19.6% in comparison to 22.2% for the untreated cells) whereas sodium butyrate treatments caused a stronger, statistically significant reduction of lentivirus transduction efficacy (17.6% in comparison to 22.2% for the untreated cells). L-lactate treatment of HeLa cells resulted in the highest reduction of lentivirus transduction efficacy as we detected only 17% of responding cells. Our results suggest that both lactate stimulation pathways via surface HCA1 and intracellular action via HDAC inhibition and chromatin rearrangement are responsible for the observed effects of lactate on the enhancement of DNA-PKcs nuclear localization and the inhibition of lentivirus transduction.

### 2.6. Enhancement of DNA-PKcs Nuclear Translocation by Intra/Extracellular Lactate Is Related to Monocarboxylate Transporters Activity

**Figure 6:**
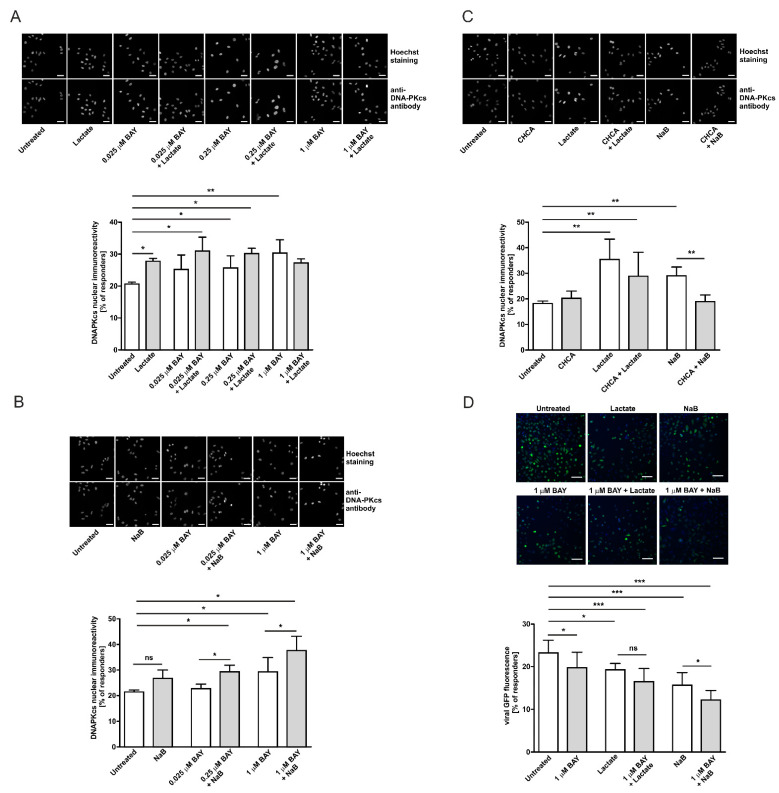
Enhancement of DNA-PKcs nuclear translocation by intra/extracellular lactate is related to monocarboxylate transporter activity.(**A**) HeLa cells were treated with increasing concentrations of BAY-8002 (0–1 µM) for 1h prior to 24 h incubation in the presence or absence of 20 mM L-lactate. The graphs display the percentage of high DNA-PKcs immunoreactive cells (responders) whose fluorescence overcame the threshold of mean fluorescence set on untreated cells. Data are presented as mean ± SD (n≥3) (**B**) HeLa cells were treated with BAY-8002 (0.025 µM or 1 µM) for 1h prior to 24 h incubation in the presence or absence of 0.5 mM sodium butyrate. The graphs display the percentage of high DNA-PKcs immunoreactive cells (responders) whose fluorescence overcame the threshold of mean fluorescence set on untreated cells. Data are presented as mean ± SD (n = 3). (**C**) HeLa cells were treated with CHCA at 2 mM concentration for 1 h prior to 24 h incubation in the presence or absence of 20 mM L-lactate or 0.5 mM sodium butyrate. The graphs display the percentage of high DNA-PKcs immunoreactive cells (responders) whose fluorescence overcame the threshold of mean fluorescence set on untreated cells. Data are presented as mean ± SD (n ≥ 3). (**D**) HeLa cells were pretreated either with BAY-8002 1 µM for 1 h or left untreated prior to 24 h incubation in the presence or absence of 20 mM L-lactate or 0.5 mM sodium butyrate. The next day, cells were infected with GFP Control Lentiviral Particles at MOI = 5 for 24 h before changing the medium. After 48 h, infected cells were stained with Hoechst 33342 and immediately analyzed using an ArrayScan VTI HCS Reader. The graphs show the percentage of high copGFP expressing HeLa cells (responders) whose fluorescence overcame the threshold of mean fluorescence set on untreated cells. Data are presented as mean ± SD (n = 3). Images showed representative microscopic areas for the particular treatment from the same experiment. The scale bar is 50 µm. * *p* < 0.05, ** *p* < 0.01 and *** *p* < 0.001; not significant, ns.

According to a preclinical study of Quanz and colleagues, the treatment of human Burkitt lymphoma tumor-bearing mice with novel and potent dual MCT1/MCT2 inhibitor BAY-8002, resulted in the intratumor accumulation of lactate [30]. Thus, in the following step we decided to evaluate the role of cell-originated lactate (formed during anaerobic glycolysis) in the nuclear translocation of DNA-PKcs. At the lowest BAY-8002 concentrations tested (0.025 µM) we observed slightly enhanced DNA-PKcs nuclear localization in 24% of the cells compared to 20.4% of the untreated cells (Figure 6A). The treatment of HeLa cells with the higher concentrations of the inhibitor (0.25–1 µM) further increased the percentage of highly DNA-PKcs immunoreactive cells by between 27 and 29% of the examined cells. Thus, the application of BAY-8002 resulted in a concentration-dependent increase of the DNA-PKcs level in the nucleus that was statistically significant in the experiments with higher doses of the compound. Interestingly, the concomitant treatment of cells with the inhibitor and 20 mM L-lactate increased the percentage of responding cells to the higher extent in comparison to the inhibitor alone. The highest percentage of responders (31.6%) was observed at 0.25 µM BAY-8002 plus 20 mM L-lactate and the effect of 20 mM L-lactate supplementation diminished along with the inhibitor concentration increase. At the highest BAY-8002 concentration tested, the extent of increase in DNA-PKcs nuclear localization after extracellular treatment with 20 mM L-lactate was comparable with the effect of the 1 µM BAY-8002 treatment (29% vs. 28.9%). In the next step, we performed similar experiments using 0.5 mM sodium butyrate in combination with 0.025 µM and 1 µM BAY-8002 to avoid overlapping effects of the same monocarboxylate molecule (butyrate is not produced by mammalian cells but the fermentation bacteria). The combined treatment of HeLa cells with BAY-8002 and sodium butyrate accounted for their additive effects on the enhancement of DNA-PKcs nuclear translocation (Figure 6B). To further explore the involvement of MCTs in the lactate-mediated enhancement of DNA-PKcs nuclear translocation we designed an experiment to inhibit extracellular lactate influx by using a non-specific MCTs inhibitor, α-cyano-4-hydroxycinnamic acid (CHCA). For this purpose, we treated cells for 1 h with 2 mM CHCA prior to 24 h incubation with 20 mM L-lactate or 0.5 mM sodium butyrate. Although pretreatment with CHCA only partially abolished the effects of 20 mM L-lactate (but not statistically significant), the percentage of responding cells to butyrate treatment was significantly reduced by CHCA (Figure 6C). These results provide functional evidence on the MCTs-dependent gating of lactate flux and its intracellular bioavailability. Collectively, it is convincing that the coordinated interplay of cellular MCT1–MCT4 is indirectly responsible for the lactate-driven enhancement of DNA-PKcs nuclear localization. In the next step, we performed complementary experiments to assess the functional relevance of MCTs to the lactate-mediated repression of lentivirus transduction. Stimulation of the cells with 1 µM BAY-8002 alone significantly reduced lentivirus transduction efficacy compared to the control cells as expected (23.2% vs. 19.75% responding cells; Figure 6D). Pretreatment of the cells with 1 µM BAY-8002 prior to incubation with butyrate, but not lactate, further decreased lentivirus transduction compared to BAY-8002 alone. The effect of BAY-8002 plus butyrate on lentivirus transduction was additive and reflected complementary results on DNA-PKcs nuclear translocation. The effects of combined treatment with BAY-8002 plus lactate vs. BAY-8002 did not differ significantly.

## 3. Discussion

According to data, the eukaryotic DNA repair pathway, namely NHEJ components, can promote or restrict virus transduction/replication by directly manipulating viral nucleic acids and/or activating signaling pathways that can impact viral life cycles [12,29,31]. They have been implicated in the interaction with dsDNA viruses, ssDNA viruses, ssRNA viruses, ssRNA-RT viruses, and dsDNA-RT viruses [12]. Previously, our study demonstrated that changes in HCA1 receptor expression or the stimulation of cervical epithelial cells with receptor ligands L-lactate or 3,5-dihydroxybenzoic acid induced the nuclear translocation of DNA-PKcs, which translated into a higher DNA repair rate [2,10]. Since NHEJ is engaged in retroviral integration processes (e.g., HIV-1) and DNA-PKcs is regulated by lactate we decided to explore the possible role of lactate in retroviral transduction processes in cervical epithelial cells. Indeed, our study demonstrated that L- and D-lactate stimulated the nuclear translocation of DNA-PKcs in cervical epithelial cancer cells HeLa, CaSki, and C33A. The incubation of cells with L- and D-lactate enhanced the DNA-PKcs presence in nuclear compartments of HeLa, CaSki, and C33A cells by 63%, 41%, and 38%, respectively (Figure 1C). Interestingly, the response potency to the lactate of each cell line was inversely correlated with its cellular protein expression in resting cells, as revealed by Western blot assay (Figure 1A). In addition, while HeLa and CaSki cells responded to both lactate isomers, the most DNA-PKcs-abundant C33A cells responded only to L-lactate. These results agree with the previous reports on the lactate-driven increase of chemoresistance where C33A cells were the least prone to D-lactate stimulation [2]. Taken together, combined observations may indicate that the most DNA-PKcs-proficient cells among cervical cancer cells are less susceptible to lactate modulation due to the saturation of cellular compartments with DNA-PKcs and, thus, relatively unresponsive to novel stimuli.

Our study is the first to demonstrate that the stimulation of cervical cancer cells with lactate suppresses the transduction rate of lentiviruses. We have shown that the incubation of cervical epithelial cells with 20 mM of L- or D-lactate decreased the transduction rate of engineered retroviral vectors along with an inversely proportional DNA-PKcs nuclear increase (Figure 1C vs. Figure 2). Though the DNA-PK complex is essential for efficient lentiviral integration, we have observed that an increased amount of DNA-PKcs in the nucleus might act as a negative regulator of viral transduction. We proposed that this apparent contradiction may be due to the fact that a lactate-driven nuclear influx of DNA-PKcs may confer the unfavorable composition of the DNA–PK complex resulting in improper action at the viral cDNA integration site. It is well known that presence of linear viral DNA may also rapidly induce its circularization into genomes harboring one- or two-long terminal repeats (1-LTR and 2-LTR, respectively) [31]. These structures are believed to represent an unproductive pathway leading to the end of the viral cycle. According to Li et al., the NHEJ system is responsible for the ligation of the LTR sequences at the retroviral cDNA to produce 2-LTR circles. Interestingly, the 2-LTR circular form of the viral cDNA was absent in infected mutant cells in the NHEJ pathway [31]. Moreover, we previously reported that DNA-PKcs nuclear translocation stimulated by lactate accelerate the DNA repair rate [2,10]. Thus, it seems likely that enhanced DNA repair driven by lactate-recruited DNA-PKcs could translate into a higher rate of 2-LTR ligation over proper viral cDNA integration events. Furthermore, Zhang and co-workers showed that DNA-PKcs overexpression leads to the inhibition of HIV-1 transcription [17]. Taken together, it is conceivable that the lactate-elicited increase in nuclear DNA-PKcs could translate into defective lentiviral transduction.

**Figure 7:**
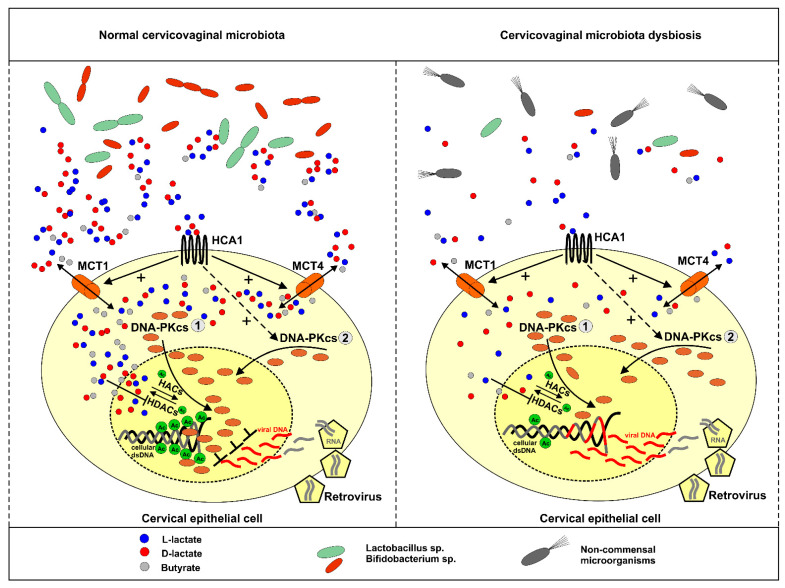
Illustration of hypothetic role of lactate in anti-lentiviral protection of female genital tract. Under normal conditions symbiotic microbiota produce L-/D-lactate and butyrate which can be imported by cervical epithelial cells through MCT1-4 transporters. In the nuclear compartment, lactate and butyrate inhibit histone deacetylases (HDACs), thus, promotes histones acetylation by histone acetylases (HACs). Acetylated histones induce chromatin relaxation and recruitment of DNA-PKcs to nucleus (1 pathway). Cervical epithelial cells bear HCA1, a specific receptor for lactate. Lactobacilli sp. derived lactate stimulates surface HCA1, induce cAMP signaling and cAMP/EPAC/PKA-dependent shuttling of DNA-PKcs to nuclear compartment (2 pathway). Excessive nuclear localization of DNA-PKcs protects cells from lentiviral (e.g., HIV-1) transduction. Cervicovaginal microbiota dysbiosis accounts for lactate shortage and insufficient anti-lentiviral protection of first-line defense epithelial cells.

To further confirm the role of DNA-PKcs in the transduction of lentiviral particles and modulatory effects of lactate on transduction efficacy, we employed an established model of glioma cells exhibiting either proficient DNA-PKcs phenotype, M059K, or deficient DNA-PKcs phenotype, M059J. As expected, M059J cells negatively immunostained with anti-DNA-PKcs antibodies were profoundly defective in lentivirus transduction, and only 8.5% of the cell population was infected (Figure 3C). Contrarily, the DNA-PKcs-proficient counterpart, M059K, was susceptible to the lactate-driven enhancement of DNA-PKcs nuclear localization and the inhibition of lentiviral transduction at a similar rate as cervical epithelial cells. To extend the observations on the role of DNA-PKcs in lentiviral transduction efficacy, we used a specific inhibitor, NU7441, against DNA-PKcs. Both examined cell lines HeLa and M059K treated with NU7441 showed a diminished transduction rate of lentivirus as described earlier (Figure 4). Taken together, our presented model of lactate-modulated lentiviral interaction with cervical epithelial cells are in line with the previous observations emphasizing the essential role of DNA-PKcs in lentiviral transduction [15,17,29]. In the previous study, we have shown that lactate-induced DNA repair enhancement is regulated by the HCA1 and HDAC activity, as lactate receptor down-regulation or restriction of lactate flux by CHCA, a pan-MCT inhibitor, notably affected DNA repair efficacy [2]. Complimentary experiments performed using DHBA and sodium butyrate showed that the observed modulatory effects of lactate on DNA-PKcs nuclear localization are related to lactate-specific activity and are dependent, in part, on its extracellular (through stimulation of HCA1) and intracellular activity via the inhibition of HDAC (Figure 5A). In this mechanism, L- and D-lactate (including sodium butyrate) are actively transported across the cell membrane by MCTs to intracellular compartments, leading to the inhibition of HDACs. This inhibition results in the hyperacetylation of histones H3 and H4, chromatin relaxation, and the attraction/activation of DNA-PKcs to the nucleus [2,32]. The other pathway involves HCA1 activation. LAB-derived lactate stimulates surface HCA1, leading to the activation of the receptor-coupled inhibitory Gi protein and drop in cAMP synthesis. According to the observations of Huston et al., cAMP serves as an agonist for EPAC and PKA, which have been shown to control the DNA-PK nuclear exit through antagonistic EPAC/PKA interplay [33]. Indeed, detailed experiments using the HCA1 agonist revealed that the net sum of particular treatment effects of DHBA and sodium butyrate on DNA-PKcs constituted a total net effect observed for 20 mM L-lactate (hypothetic role of HCA1 and HDAC in lactate-driven inhibition of lentiviral transduction is illustrated in Figure 7). As expected, the effects of DHBA and sodium butyrate treatments on DNA-PKcs translated into a reverse pattern of the response in the experiments assessing lentiviral transduction efficacy. Collectively, these experiments confirmed the role and showed the mechanism of the intrinsic lactate activity in the lactate-driven enhancement of DNA-PKcs nuclear localization and the inhibition of lentiviral transduction. Cellular lactate uptake and efflux depend on the activity of monocarboxylate transporters (MCTs 1–4), directed by substrate and proton concentration gradients [9]. Previously, we have shown that HeLa cells express MCT1, MCT2, and MCT4 transporters [2]. Furthermore, the expression of the latter one was linked with lactate receptor HCA1 expression as HCA1 silencing negatively affected the mRNA level of MCT4 [2]. Recently, MCT1 inhibition has been recognized as an attractive therapeutic strategy against cancers since the blockade of lactate transport was found to affect tumor growth either through the accumulation of intracellular lactate formed during anaerobic glycolysis or the restriction of lactate uptake and, thus, the fueling of cell oxidative phosphorylation [30]. Our study is the first to demonstrate that the inhibition of L-lactate flux with MCT1/MCT2 inhibitor BAY-8002 induced DNA-PKcs nuclear translocation, presumably via the accumulation of endogenous lactate. According to the Quanz study, BAY-8002 inhibits L-lactate efflux via MCT1 with an EC50 = 77 nM in MCT1-expressing Raji cells, and maximum inhibition was observed at 1 µM BAY-8002 [30]. Indeed, in our study along with the MCT1 inhibitor doses we observed a concentration-dependent increase in DNA-PKcs nuclear translocation up to 1 µM BAY-8002. Interestingly, the incubation of cells with 20 mM L-lactate followed by between 0.025 and 0.25 µM BAY-8002-treatment evoked a further increase in DNA-PKcs nuclear translocation. Since BAY-8002 is a highly selective MCT1/2 inhibitor, this observation would indicate an active role of the MCT4 transporter in lactate uptake. Indeed, as a consequence of intracellular lactate build-up, due to BAY-8002 treatment, the increase of MCT4-expression characteristic for hypoxic conditions through lactate-stabilized HIF1α was expected [30]. Moreover, functional studies on monocarboxylate transport via MCT4 by Contreras-Baeza and colleagues revealed that MCT4-bearing cells reverse to lactate importers in a microenvironment with lactate above 13 mM [34]. Thus, as a consequence of MCT1/MCT4 and lactate interplay we observed an increase in DNA-PKcs nuclear localization in 32% of cells concomitantly treated with 0.25 µM BAY-8002 plus 20 mM L-lactate (Figure 6A). Consistently, we observed combined effects of BAY-8002-mediated intracellular lactate build-up and MCT4-driven lactate influx until increasing BAY-8002 concentrations at 1 µM elicited intracellular lactate saturation or lactate reached its cellular capacity. To extend the study on the role of MCT4 in the lactate-mediated enhancement of DNA-PKcs nuclear translocation, we designed an experiment to inhibit extracellular lactate influx by using non-specific MCTs inhibitor CHCA. CHCA is a small molecule that does not enter the cell and its inhibitory effect is dependent on interactions with MCTs proteins accessible from the outside of the cell [35]. It is known that CHCA can inhibit different MCT isoforms including MCT4 but at much a higher concentration (Ki = 0.99 mM) [9]. In these experiments, the pan-MCT inhibitor significantly decreased the effects of sodium butyrate and partially reversed the effect of L-lactate. Collectively, these results provided functional evidence on the MCTs-dependent control on lactate flux and its cellular fate and intracellular activity. Finally, we performed complementary experiments to assess the effects of combined treatments with BAY-8002 and lactate or BAY-8002 and butyrate on lentiviral transduction efficacy. As expected, the effects of treatments with BAY-8002 and dual treatments with BAY-8002 and lactate or butyrate diminished lentiviral transduction efficacy and were inversely correlated with observed effects of DNA-PKcs nuclear translocation. Collectively, these results suggest that MCTs may play an important role in cell susceptibility to lentiviral transduction. Moreover, the latter experiments showed novel inhibitory potential of BAY-8002 against retroviral infections through the lactate-mediated enhancement of DNA-PKcs nuclear localization. Unfortunately, we could not replicate complementary experiments to test the effects of CHCA on the lactate-driven suppression of lentiviral transduction due to observed huge non-specific inhibitory effects on viral transduction efficacy.

The mucosal epithelial cells of the female genital tract, along with lactic acid symbiotic bacteria, serve as a first-line barrier against invasive pathogens. The present study reports a novel biological activity of LAB-originated lactate, specifically in the suppression of the transduction efficacy of lentivirus in cervical cancer cells through the modulation of DNA-PKcs cellular localization. Our study demonstrated that both lactate isomers enhanced the presence of DNA-PKcs in the nuclear compartments of HeLa, CaSki, and C33A cells over 60%, which translated into an almost 40% lower lentiviral transduction rate.

This preliminary study was aimed at gaining novel insight into the possible role of DNA-PKcs in the modulation of viral transduction in a lactate-rich environment. Since we could observe enhanced nuclear localization of DNA-PKcs upon lactate treatment we tentatively assumed its modulatory action in the nucleus and the restriction of lentivirus transduction at the integration level. This assumption was supported by numerous reports emphasizing the crucial role of DNA-PKcs in driving viral cDNA to genome integration or to unproductive pathway events. Similar to other studies which use engineered retroviral vectors and the technique of high content analysis, we could examine the retroviral infection of epithelial cells at the single-cell level. On the other hand, with such an approach, we could neither exactly define which step of lentiviral particles infection was blocked by the lactate treatment nor quantitate the levels of integrated viral DNA. Further in-depth studies are needed to elucidate whether, and how, lactate contributes to the restriction of viral infections. Moreover, it would be fascinating to determine the influence of lactate on the integration of HIV-based vectors, especially in the context of recent research suggesting that cervical epithelial cells can be effectively infected with HIV-1.

Importantly, the present study provides new insight into the role of microorganism–mammalian cell interactions in the female genital tract and demonstrates a novel mechanism underlying the regulation of cellular resistance to viruses.

## 4. Materials and Methods

### 4.1. Chemicals

All chemicals were purchased from Sigma-Aldrich (St. Louis, MO, USA) unless otherwise stated. BAY-8002 was purchased from MedChemExpress LLC (Monmouth Junction, NJ, USA).

### 4.2. Cell Culture

The HeLa, Ca Ski, C33A human cervical cancer cell lines, and M059K and M059J glioma-derived cell lines, were purchased from the American Type Culture Collection (ATCC, Manassas, VA, USA). HeLa cells, M059K, and M059J cells were cultured in DMEM, and Ca Ski and C33A cells were cultured in RPMI medium (Life Technologies, Carlsbad, CA, USA) supplemented with 10% fetal bovine serum (PAA Laboratories GmbH, Pasching, Austria) and antibiotics (Life Technologies) at 37 °C in a humidified atmosphere containing 5% CO_2_. The cells were routinely tested for mycoplasma contamination and were passaged every 3 days using TrypLE Express (Life Technologies).

### 4.3. DNA-PKcs Immunocytochemistry

Cells grown on a 96-well plate were washed with PBS and fixedwith 4% formaldehyde for 20 min, permeabilized (0.25% Triton X-100 in PBS, 10 min), and blocked (3% BSA in PBST, 30 min) before incubation in an anti-DNA-PKcs antibody (sc-9051, Santa Cruz Biotechnology, Inc., Dallas, Texas, USA) as the primary antibody overnight at 4 °C. Primary antibody binding was visualized using an Alexa Fluor 488-conjugated goat anti-rabbit antibody (Life Technologies) followed by nuclear staining with 2 μg/mL Hoechst 33,342 for 20 min. Images were acquired using an ArrayScan VTI HCS Reader equipped with a 20× objective and analyzed using Molecular Translocation Bioapplication V3 software (250 cells/well). Cells exhibiting immunofluorescence higher than the average population were considered to be responders. Each experiment was performed in four replicates.

### 4.4. Western Blot Analysis

Cells grown at 70% confluence were harvested using a cell scraper, centrifuged, and lysed with RIPA buffer supplemented with a protease inhibitor cocktail (Roche Diagnostics GmbH, Mannheim, Germany). Cellular extracts were separated by SDS PAGE (NuPAGE gradient gel 4–12%, Life Technologies) and transferred into a nitrocellulose membrane. After 1 h of blocking (5% defatted milk in PBST) and overnight incubation at 4 °C with primary antibodies, anti-DNA-Pkcs or anti-β-actin (sc-9051 and sc-1616, respectively, Santa Cruz Biotechnology, Inc.) membranes were washed with PBS and incubated in an HRP-conjugated secondary antibody (Dako, Ely, UK) for 1 h at RT. Antibody binding was visualized via the chemiluminescent method using NOVA 2.0 chemicals (Cyanagen, Bologna, Italy). Chemiluminescence signals were captured and quantified using a G:BOX gel imager (Syngene, Cambridge, UK).

### 4.5. Single Round Transduction Assay

Cells grown on a 384-well plate were transduced with the copGFP Control Lentiviral Particles, VSV-G pseudotyped (sc-108084, Santa Cruz Biotechnology, Inc.) at MOI = 5 (optimal transduction efficacy for HeLa cells) in the presence of 5 μg/mL of polybrene (Sigma, St. Louis, MO, USA) for 24 h before changing the medium. After 48 h, the infected cells were washed with PBS, stained with 5 μg/mL Hoechst 33342 for 60 min, and live cells were immediately analyzed using an ArrayScan VTI HCS Reader equipped with a 10× objective. Images were analyzed using Molecular Translocation Bioapplication V3 software (2500 cells/well). The efficacy of the transduction of cells with lentivirus was approximately 95%. Cells exhibiting copGFP fluorescence higher than the average population were considered to be responders. Each experiment was performed in four replicates and repeated at least three times.

### 4.6. Population Characterization (Responders) Principles

Molecular Translocation Bioapplication V3 software (Cellomics, Inc., Pittsburgh, PA, USA) was used to characterize populations of the treated and untreated cells in relation to their fluorescence. For this purpose, the mean fluorescence of the cells measured in the untreated population was set as the threshold value. In the next step, such a defined threshold was implemented in software settings and used for analysis of the cell populations distribution. The percentage of cells whose fluorescence overcame the threshold was reported as responders (or high fluorescence cells). With the manual setting of the threshold, we reportedbetween 19 and 25% of respondersin the untreated cells population for each run and a higher or lower percentage of responders in the populations of the treated cells. By using this approach, we were able to avoid run-to-run variations due to different illumination conditions, fluorophore amounts, or changes in cell conditions.

### 4.7. Statistical Analysis

The experiments were repeated at least three times, each of which was conducted in 3–6 replicates. The data are presented as the means ± SD. GraphPad Prism software v. 5.02 was used to analyze and plot the data. Statistical significance was evaluated using one-way ANOVA followed by Tukey’s test or Student’s *t*-test.

## Data Availability

The data presented in this study are available upon request from the corresponding author.

## Abbreviations

HCA1, hydroxycarboxylic acid receptor 1; GPCR, G-protein coupled receptor; FGT, female genital tract; HDAC, histone deacetylase; DHBA, dihydroxybenzoic acid, DNA-PKcs, DNA-dependent protein kinase catalytic subunit; NHEJ, nonhomologous end joining; PKC, protein kinase C;PKA, protein kinase A; cAMP, Cyclic adenosine monophosphate; EPAC, exchange protein activated by cAMP;ADV, Adenovirus; HPV, Human papillomavirus; HSV-1, Herpes simplex virus 1; EBV, Epstein Barr virus; HIV, Human immunodeficiency virus;HTLV, Human T-cell leukemia virus type; LTR, long terminal repeat; MCT, monocarboxylate transporter.
